# Meaning in Life, Social Support, and Happiness in Older Adults: The Moderating Role of Daily Activities

**DOI:** 10.3390/healthcare14152412

**Published:** 2026-08-05

**Authors:** Arta Dalipi Shabani, Suzanne Denieffe, Margaret Denny

**Affiliations:** 1 Faculty of Health Sciences, University of Maribor, 2000 Maribor, Slovenia; denny.margaret@gmail.com; 2 Faculty of Medicine, University ‘’Fehmi Agani’’, 50000 Gjakova, Kosovo; 3 Faculty of Arts and Humanities, South East Technological University, X91 K0EK Waterford, Ireland; suzanne.denieffe@setu.ie

**Keywords:** healthy ageing, psychosocial well-being, social support, functional ability, older adults, community care, person-centred care

## Abstract

**Highlights:**

**What are the main findings?**

- Meaning in life and perceived social support were significant positive predictors of happiness among older adults, confirming the central role of psychosocial resources in healthy ageing.
- Daily functioning (GARS) did not directly predict happiness but significantly moderated the relationships between meaning in life, social support, and well-being, indicating that psychosocial resources become more important as functional limitations increase.

**What are the implications of the main findings?**

- Interventions for older adults should adopt an integrated approach that simultaneously strengthens meaning in life, enhances social support, and promotes engagement in daily activities that are adapted to their functional abilities.
- Community-based and healthcare services should routinely assess both psychosocial well-being and functional capacity to develop person-centred strategies that improve happiness and quality of life in ageing populations.

**Abstract:**

**Background:** Psychological well-being, meaning in life, social support, and daily activities are well-documented factors that significantly affect the psychological well-being of older adults. Nevertheless, research on the interactions among these variables and their collective impact on happiness is limited. The objective of this study was to examine the effects of meaning in life and social support on the overall happiness of older adults. **Methods:** A quantitative study employing a descriptive-analytic, cross-sectional design was conducted with 221 participants aged 65 and older who attended day-care centres in Pristina. The study used four validated instruments: the Pemberton Happiness Index (PHI), the Meaning in Life Questionnaire (MLQ), the Multidimensional Perceived Social Support Scale (MSPSS), and the Groningen Activity Restriction Scale (GARS). The statistical analyses included linear regression and interaction effects, with a significance level of *p* < 0.05. **Results:** Meaning in life (β = 0.614, *p* = 0.039) and social support (β = 7.695, *p* = 0.012) were significant positive predictors of happiness. GARS was not shown to have a direct main effect but emerged as a significant moderating factor. Interaction effects of MLQ × GARS (*p* = 0.037) and MSPSS × GARS (*p* < 0.001) showed that functional limitations strengthen the positive impact of meaning in life and social support on subjective well-being. **Conclusions:** Meaning in life and social support are important psychosocial determinants of well-being among older adults, particularly in the presence of functional limitations. The findings highlight the importance of integrated, person-centred approaches within community and healthcare services that address psychosocial well-being alongside functional capacity. Routine assessment of psychosocial and functional needs may support more effective interventions aimed at improving quality of life and healthy ageing among older adults.

## 1. Introduction

The ageing of populations is a major demographic shift globally and is likely to have serious effects on health and social care systems. Current estimates indicate that the population aged 60 years and over will more than double by 2050, placing additional demands on both healthcare resources and social support systems [1,2]. Not only does increased longevity indicate significant improvements in public health, but it also suggests that chronic disease, functional limitations, and social vulnerability are increasing.

Current research has begun to conceptualise well-being as multidimensional, comprising hedonistic well-being (happiness, life satisfaction) and eudaimonic well-being (a sense of meaning, purpose, and personal growth) [3,4]. Meaning in life is considered a central psychological resource, and individuals who report a strong sense of meaning in their lives exhibit higher levels of psychological well-being, lower depressive symptoms, and greater resilience [5,6,7]. The concepts of happiness, well-being, quality of life, and life satisfaction are interrelated but denote distinct constructs. In the current study, happiness serves as the primary outcome and is assessed using the Pemberton Happiness Index (PHI) [8]. Well-being is conceptualised as a broader notion encompassing both hedonic and eudaimonic dimensions; quality of life additionally incorporates functional, social, and environmental aspects; and life satisfaction refers to an individual’s overall cognitive assessment of their life circumstances. While these terms are referenced in connection with existing literature, all hypotheses and analyses presented in this study are specifically centred on happiness as the dependent variable.

Social support has been identified as a key determinant of well-being in older individuals, contributing to improved mental and physical health and enhancing sense of belonging and life satisfaction [9,10,11,12]. Older adults engaged in social networks are less likely to feel lonely, have a lower risk of becoming depressed, and report higher quality of life [13].

Functional ability, a person’s capacity to perform everyday activities and tasks, is similarly central to well-being in later life. It strongly correlates with healthcare use, treatment adherence, and self-management of chronic conditions [1,2], and directly shapes overall quality of life, as improvements in functional capacity can enhance well-being while declines can undermine a person’s sense of fulfilment and happiness. As expected, functional decline correlates with age and is often assumed to reduce overall quality of life; however, several studies suggest that the rate of functional decline may not affect an individual’s overall well-being as directly as expected [14,15]. This apparent paradox highlights the need to further examine how physical, psychological, and social factors interact in shaping well-being in older age.

The theoretical basis for the present study draws on two complementary frameworks. The stress-buffering model [16] posits that psychosocial resources including meaning in life and social support exert stronger protective effects on well-being as an individual becomes more vulnerable, that is, under conditions of elevated stress or functional constraint. Complementing this view, the Activity Theory of Ageing [17,18] proposes that continued engagement in meaningful and social activities is essential for sustaining psychological well-being in later life, with functional ability determining the degree to which older adults can participate in such activities. Individuals with higher functional capacity may derive happiness from a broader range of sources, making the contribution of meaning in life and social support comparatively less decisive, whereas those with lower functional ability are more dependent on internal psychological resources and relational ties, a dependence that may amplify the associations between meaning in life, social support, and happiness. These two frameworks generate the prediction that functional ability moderates the relationship between psychosocial resources and happiness, such that these associations are stronger among individuals with lower functional capacity.

Although previous research has examined the independent contributions of meaning in life and social support to happiness and life satisfaction among older adults [7,9,10,19,20], the moderating role of functional ability in these relationships has received limited empirical attention. The few studies that have investigated moderation in this context have predominantly used broader indicators such as physical health or self-reported health as moderators of life satisfaction and psychological well-being [13,20], rather than daily functional capacity specifically. Evidence from South-Eastern European contexts is likewise scarce. The present study addresses these gaps by concurrently testing the moderating role of functional ability on two distinct psychosocial predictors of happiness, meaning in life and social support within a single community-based sample of older adults in Kosovo.

A significant theoretical decision in this study concerns the conceptualization of functional ability as a moderator rather than a mediator in the relationship between psychosocial resources and happiness. Conceptualising functional ability as a mediator would imply that meaning in life and social support influence happiness indirectly through their impact on functional ability for instance, that increased meaning in life enhances physical functioning, which in turn produces greater happiness. While such pathways are theoretically plausible, the cross-sectional design of this study does not allow for testing sequential causal chains. Instead, functional ability is treated as a pre-existing, contextual condition that delineates the circumstances under which psychosocial resources operate, consistent with the distinction articulated by [21] between moderators, which affect the direction or strength of a relationship, and mediators, which explain the mechanism underlying it. In this study, functional ability is assessed using the Groningen Activity Restriction Scale (GARS), a stable indicator of disability and independence in activities of daily living, making it theoretically more appropriate to treat it as a boundary condition (moderator) than as a process variable (mediator) within the current design.

Based on the theoretical frameworks and empirical evidence reviewed above, the following hypotheses are proposed:

**Hypothesis** **1.** *Meaning in life will be positively associated with happiness in older adults, such that higher levels of meaning in life predict higher levels of happiness.*

**Hypothesis** **2.** *Social support will be positively associated with happiness in older adults, such that higher levels of perceived social support predict higher levels of happiness.*

**Hypothesis** **3.** *Functional ability will moderate the association between meaning in life and happiness, such that this association will be stronger among older adults with lower functional ability.*

**Hypothesis** **4.** *Functional ability will moderate the association between social support and happiness, such that this association will be stronger among older adults with lower functional ability.*

**Figure 1:**
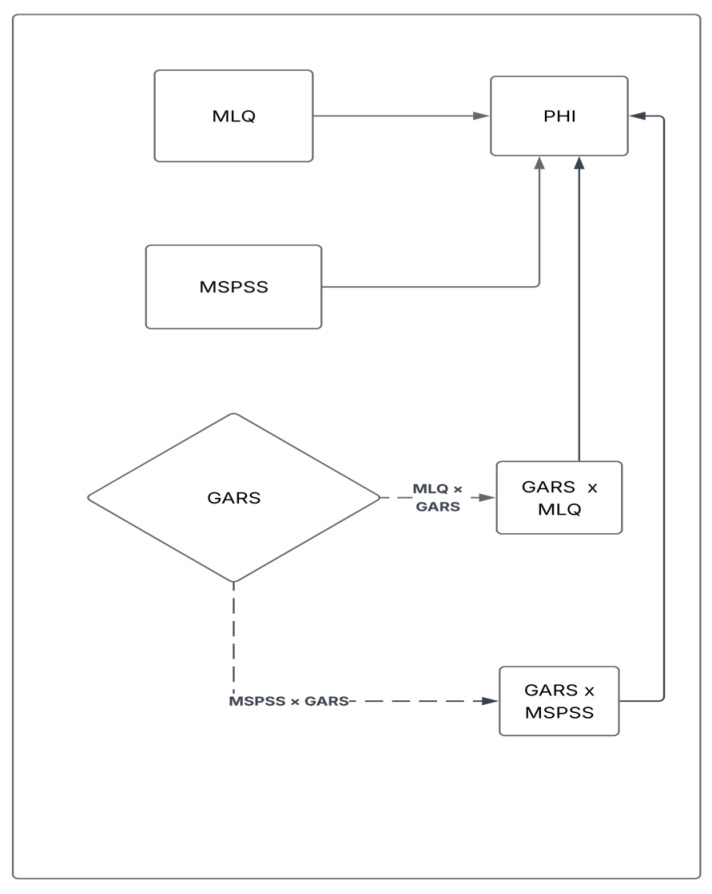
Conceptual model illustrating the relationships between meaning in life, social support, and well-being, with functional ability as a moderating variable.

Figure 1 depicts the hypothesised moderation model. Direct paths are shown from meaning in life and social support to happiness. Functional ability is proposed as a moderator of both relationships, such that the associations between meaning in life and happiness and between social support and happiness vary with participants’ level of functional ability.

The proposed moderation model is established upon two complementary theoretical frameworks. The stress-buffering model, developed by [17], posits that psychosocial resources, such as a sense of meaning in life and social support, become particularly vital in the context of stress or functional limitations. In a similar vein, the Activity Theory of Ageing, as articulated by [17], emphasises the importance of engaging in meaningful, social activities to promote well-being in later life. Given that functional ability influences a person’s capacity to engage in such activities, older adults with diminished functional ability may rely more heavily on psychosocial resources, such as meaning in life and social support, to sustain their happiness. Collectively, these frameworks offer a robust theoretical foundation for examining functional ability as a moderator of the relationships between psychosocial resources and happiness.

Functional limitations may strengthen the associations among meaning in life, social support, and happiness by necessitating greater reliance on psychosocial resources when daily functioning is impaired; conversely, they may also weaken these associations by limiting opportunities for meaningful social interaction [22]. Informed by the stress-buffering model and resource-dependency perspectives on ageing [21], this study posits that diminished functional ability will strengthen the positive relationships between meaning in life, social support, and happiness though these effects may not be uniform across individuals. Building on this integrative perspective, the present study examines the direct effects of meaning in life and social support on happiness, as well as the moderating role of functional ability, among community-dwelling older adults in Kosovo.

These effects may not be uniform across individuals. Functional ability may influence the extent to which individuals benefit from psychosocial resources, as limitations in daily activities can reduce the positive impact of social support and meaning in life. Building on these perspectives, the present study aimed to examine the direct effects of meaning in life and social support on well-being, as well as the moderating role of functional ability among community-dwelling older adults.

## 2. Materials and Methods

### 2.1. Study Design and Setting

This study employed a quantitative, cross-sectional design to examine the relationships between meaning in life, social support, daily activities, and happiness among older adults living in the municipality of Pristina. Following a structured, standardised research protocol, data were collected in day centres for older adults operated by the Department of Social Welfare in Pristina. The design aligns with contemporary public health and gerontological research approaches, aiming to capture population-level associations using validated psychometric instruments. The methodological framework was informed by previous epidemiological studies in ageing populations.

### 2.2. Participants

The study’s target population consisted of older adults aged 65 years and older residing within the community. Two hundred and twenty-one individuals (*N* = 221) were recruited from day centres for older adults across Pristina using purposive sampling. Purposive sampling was deemed appropriate, as this study required participants who met specific inclusion and exclusion criteria. All eligible individuals attending the centres during the study period were invited to participate. Participants were recruited from older adult day-care centres in Pristina, Kosovo, using purposive sampling. Although this approach ensured access to eligible community-dwelling older adults, it may limit representativeness, as day-care users are typically more socially engaged and may differ in functional ability and access to social support compared with non-users.

Cognitive ability was not formally evaluated through a standardised screening instrument, as it was presumed that persons attending day-care centres were functionally independent and capable of participating in activities of daily living. Nevertheless, individuals who were unable to complete the questionnaire independently, due to physical limitations, cognitive difficulties, or literacy challenges, were excluded from the study. Staff members at the day-care centres assisted in identifying potential participants and in recognising persons who might encounter difficulties with self-completion. Purposive sampling was employed to ensure that participants met the key inclusion criteria for the study objectives: specifically, they were required to be aged 65 years or older, reside in the community, and be able to complete the questionnaire independently. Day-care centres were selected as recruitment sites due to their accessibility to community-dwelling older adults with diverse levels of functional ability, which serves as the primary moderating variable in this study. Nonetheless, it is acknowledged that exclusive recruitment from day-care centres may limit the representativeness of the sample, as attendees of these services are likely to exhibit higher levels of social engagement than older adults who do not use community services.

Eligible participants were community-dwelling adults aged 65 years or older who were able to independently complete the study questionnaires and provide informed consent. Individuals who appeared unable to complete the questionnaire independently were not recruited.

Participants were given information on the study’s purpose and procedures. Completion and return of the questionnaire signified informed consent, ensuring voluntary and anonymous participation.

The target sample size was determined based on recommendations for conducting moderation analyses using multiple regression. According to the guidelines proposed by [23], a minimum of 100 to 150 participants is generally necessary to achieve adequate statistical power (1 − β = 0.80) for detecting medium-sized interaction effects (f^2^ = 0.15) in hierarchical regression models that include up to six predictors, utilising a significance level of α = 0.05. Considering this framework, a sample size of 200 participants was deemed appropriate. A post hoc power analysis performed using G*Power (v. 3.1) [24] confirmed that the final sample size of 221 participants provided adequate statistical power, exceeding 0.95, to detect the specified interaction effects.

### 2.3. Data Sources and Measurement

Data were collected using a structured, self-administered questionnaire comprising sociodemographic items and three validated scales assessing key study constructs: happiness, meaning in life, and daily activities.

All instruments were administered in Albanian. As no validated Albanian versions of the PHI, MLQ, MSPSS, or GARS were available, all instruments were translated for this study. A forward–backward translation procedure was applied. The instruments were first translated into Albanian by a bilingual researcher and subsequently back-translated into English by an independent bilingual translator. Discrepancies were resolved through discussion, and items were reviewed for semantic and conceptual equivalence by bilingual experts to ensure clarity and cultural appropriateness.

### 2.4. Instruments

Pemberton Happiness Index (PHI)—Happiness was measured using the Pemberton Happiness Index (PHI), an integrative instrument that assesses remembered and experienced well-being. The scale includes 11 items rated on an 11-point Likert scale (0–10) and 10 dichotomous items capturing daily positive and negative experiences. The PHI generates three outcomes: remembered well-being, experienced well-being, and an overall happiness index. Previous studies report robust internal consistency (α = 0.82–0.83) and cross-cultural validity [8].

Meaning in Life Questionnaire (MLQ)—Meaning in life was assessed using the Meaning in Life Questionnaire (MLQ) [25]. The MLQ consists of 10 items divided into two subscales: Presence of Meaning and Search for Meaning. Items are scored from 1 (“absolutely untrue”) to 7 (“absolutely true”), with total scores ranging from 5 to 35. The MLQ demonstrates strong psychometric performance, including adequate convergent and discriminant validity, and high internal consistency (α = 0.82–0.88).

Multidimensional Scale of Perceived Social Support (MSPSS)—Perceived social support was assessed using the Multidimensional Scale of Perceived Social Support (MSPSS) [26], a 12-item self-report instrument designed to measure perceived support from family, friends, and a significant other. The scale uses a 7-point Likert response format ranging from “very strongly disagree” to “very strongly agree.” Higher scores indicate higher levels of perceived social support. The MSPSS has demonstrated strong psychometric properties, including high internal consistency reliability, as evidenced by Cronbach’s alpha coefficients reported in previous studies [27].

Groningen Activity Restriction Scale (GARS)—Daily activities and functional ability were measured using the Groningen Activity Restriction Scale (GARS), a validated instrument assessing disability in activities of daily living (ADL) and instrumental activities of daily living (IADL) [28]. The scale contains 18 items (11 ADL, 7 IADL), each rated on a 4-point scale ranging from 1 (“no difficulty”) to 4 (“unable to perform independently”). Total scores range from 18 (no disability) to 72 (maximum disability). The GARS has demonstrated strong reliability and validity in older adult populations [28,29].

The reliability analysis obtained positive results indicating an acceptable to high level of internal consistency for each of the measures included in the study. The Cronbach’s Alpha test for the different measures demonstrated a high level of reliability: Pemberton Happiness Index (PHI) = 0.857, Meaning in Life Questionnaire (MLQ) = 0.816, Multidimensional Scale of Perceived Social Support (MSPSS) = 0.916 and Groningen Activity Restriction Scale (GARS) = 0.934. The results of the analysis indicate that the instruments were reliable and appropriate for this study.

All instruments were scored according to their standard scoring procedures as specified in the original validation studies. For the PHI, higher scores indicate greater overall happiness, and composite scores were calculated using the established algorithm that combines remembered and experienced well-being; no reverse-coding adjustments beyond the instrument’s predefined structure were required. For the MLQ and MSPSS, higher scores reflect greater meaning in life and perceived social support, respectively. For the GARS, higher scores indicate greater functional limitations (higher disability), and thus lower functional ability. Accordingly, in regression analyses, positive coefficients for PHI, MLQ, and MSPSS represent beneficial associations, whereas positive coefficients for GARS indicate greater disability. These scoring conventions were consistently applied in all statistical analyses and interpretation of results.

### 2.5. Statistical Analysis

**Figure A1:**
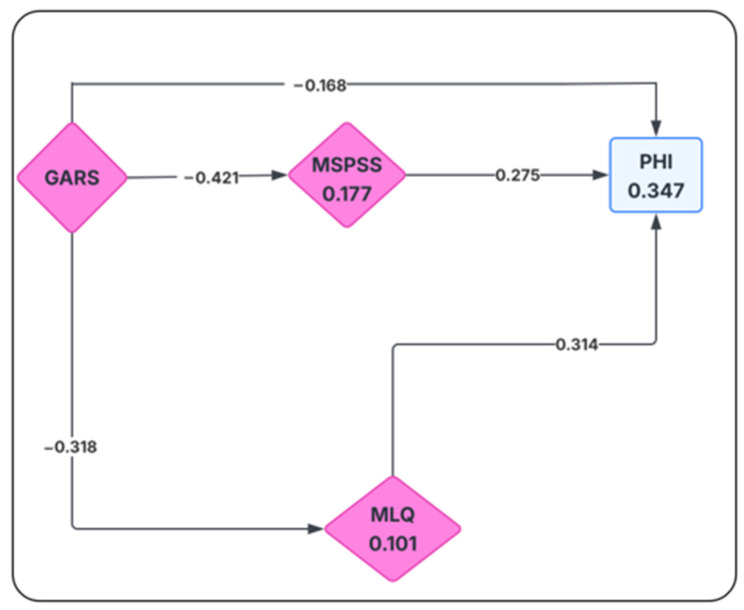
Structural equation modelling.

Data management and preliminary analyses, including descriptive statistics and reliability coefficients, were conducted using IBM SPSS Statistics (v.29). Hierarchical multiple regression analyses, including moderation models, were also performed in SPSS (v.29). STATA (v.18) was used for supplementary regression diagnostics tests, as these procedures are not directly integrated within the SPSS (v.29) analytical workflow. SmartPLS (v.4.1) was initially considered for partial least squares structural equation modelling (PLS-SEM); however, based on the study’s sample size and its primarily hypothesis-driven, confirmatory orientation, hierarchical regression was deemed the most appropriate primary analytic approach. Accordingly, SmartPLS (v. 4.1.) results are reported in Figure A1 as supplementary analyses. Conceptual diagrams were developed using Lucidchart (v. 2.1)

To ensure that all assumptions for regression analyses were satisfied, diagnostic tests (normality, linearity, homoscedasticity, multicollinearity) were conducted prior to finalising the model. Sociodemographic variables were omitted from the final model based solely on the sample size; thus, this is acknowledged as a limitation. Correlation testing and multiple regression analyses were conducted to determine the relationships between variables, including Meaning in Life (MLQ), perceived social support (MSPSS) and functional ability (GARS), and their relationship to happiness. Moderation analyses were also conducted to ascertain whether daily activities moderated the relationships between Meaning in Life (MLQ), perceived social support (MSPSS), and happiness. A *p*-value of less than 0.05 was established as the level of significance.

A single multiple-variable regression model was developed that incorporated Meaning in Life (MLQ), perceived social support (MSPSS), and functional ability (GARS) as predictor variables for happiness. Interaction variables between MLQ and GARS, and between MSPSS and GARS, were also incorporated into this model to test for moderation. The data from which the interaction variables were constructed had been mean-centred prior to modelling to ensure that all regression assumptions (normality, linearity, homoscedasticity, multicollinearity) were met.

All regression analyses were conducted using hierarchical multiple regression. All continuous predictors were mean-centred prior to analysis, and interaction terms were created from these centred variables (MLQ × GARS and MSPSS × GARS) to reduce multicollinearity and improve interpretability of the interaction effects. The reported coefficients are unstandardized estimates. The analysis was structured in two steps: in Step 1, the main effects of MLQ, MSPSS, and GARS were entered into the model; in Step 2, the interaction terms were added to test moderation effects. Model diagnostics were performed following estimation of the full model, including interaction terms, and confirmed that the assumptions of normality, linearity, homoscedasticity, and absence of multicollinearity were adequately met.

### 2.6. Ethical Considerations

The study adhered to the ethical principles of the Declaration of Helsinki (1975, revised 2008) and the Convention on Human Rights and Biomedicine [30]. Ethical approval was obtained from the Kosovo Chamber of Nurses, Midwives and Other Health Professionals (Approval Protocol No. 1051). Additional permissions were secured from the Municipality of Pristina, Department of Social Welfare, the management of participating day centres.

Participants received an information sheet explaining the purpose of the study, procedures, confidentiality safeguards, and the voluntary nature of participation. Completion of the questionnaire was considered consent for the survey component.

## 3. Results

As can be seen in Table 1, most participants in the study were men (76.9%), while women constituted 23.1%. In terms of ethnicity, the majority were Albanians (95%), with the remainder being from the Bosniak (2.3%), Turkish (1.8%), Serbian (0.5%) and Rom (0.5%) communities.

The largest group of participants were in the 65–70-year age group (40.7%), followed by the 71–75-year age group (33%), with the 76–81-year age group at 19% and only 7.2% over 82 years old. Regarding residence, 79.6% lived in urban areas and 20.4% in rural areas.

Regarding education level, most older adults had secondary education (40.7%) or primary education (29%), while a smaller proportion had university education (15.4%) or postgraduate education (5%). Only 9.5% had no formal education. Regarding marital status, 73.3% were married, 24.4% widowed, 0.9% single, and 1.4% divorced.

Regarding religion, most participants (94.1%) were Muslim, while a small proportion (1.8%) were Christian, and 4.1% preferred not to declare. In terms of income, the majority (58.4%) reported monthly incomes between €111 and €250, indicating a low level of economic status. Only 2.3% had an income between €401 and €500, while those earning over €501 represented only 0.9% of the sample. Also, 6.3% had no monthly income, while 18.1% earned less than €110 per month.

Most older adults (*n* = 203) relied on pensions as their primary source of income, while a small number (*n* = 5) had private investments and a sizeable portion (*n* = 87) received assistance from children. On average, 5.59 people lived with family members.

The Pemberton Happiness Index (PHI) in Table A1 showed high levels of happiness and subjective well-being among older adults. For most items related to the general, hedonic, and eudaimonic elements of well-being, the means were above 7 (on a scale from 0 to 10). Regarding General Well-being, the respondents reported high levels of life satisfaction (M = 7.45) and energy (M = 7.11), with moderate variation in their responses.

Regarding eudaimonic well-being, again the mean self-satisfaction (M = 7.96) and meaning in life (M = 7.59) indicated a high measure of self-worth and purpose in life. Hedonic well-being, enjoyment of small life experiences, had a mean of 7.48, while that of negative moments was less with a mean of (5.11). Social well-being had the lowest mean, 5.86.

In positive experiences, most respondents reported positive events over the last day, while negative events occurred less frequently.

As can be seen in Table A2 of the Meaning in Life Questionnaire, participants showed a medium to high perception of meaning in life, especially in the presence of meaning.

For the Presence of Meaning items, the participants reported high means for the feeling of knowing that they know the meaning of their life (5.68) and for living with a clear purpose (5.56). The item “My life does not have a clear purpose” had a lower mean (3.33).

On the Search for Meaning the mean of the items varied from 4.06 to 4.54. This indicated that some participants were searching for meaning and purpose in their lives, while others felt more satisfied with their current sense of meaning. The standard deviations were high (1.80–1.93).

The data for the Multidimensional Scale of Perceived Social Support (MSPSS) in Table A3 indicated that perceptions of their social support were high.

In the Family domain, participants reported high means ranging from 5.74 to 5.96 for items such as help, emotional support, and the ability to discuss problems with family members. In the Friends domain, the reported mean was lower than that for family, with help means ranging from 5.00 to 5.47 and higher standard deviations (1.53 to 1.69). In the Other Significant domain, participants reported high means ranging from 5.61 to 5.98 for items related to the significant other’s existence and to providing emotional support and comfort.

The data for the Groningen Activity Restriction Scale in Table A4 showed that the participants were mostly able to perform most daily and instrumental activities fully independently but had some difficulty in the more demanding physical activities.

On the Activities of Daily Living scale, the items concerning basic activities such as dressing (1.22) and eating (1.21) indicated that most subjects can perform these tasks completely independently. The standard deviations were low (0.573–0.726).

Activities such as getting out of bed (mean 1.29) or moving about the house (mean 1.34) also suggested a high degree of independence, although some individuals required minimal assistance or a cane. Other activities, such as climbing and going downstairs (mean 1.67) and moving about outside the house (mean 1.47), showed greater difficulty than the more basic activities. The items concerning personal care, such as washing the body (mean 1.33) and the care of feet and nails (mean 1.50), showed similar results.

In the Instrumental Activities of Daily Living section, higher means for items such as preparing the evening meal (2.13) and doing the heavy home chores (2.55) indicated that the participants can do these things completely independently, but with varying degrees of difficulty, which required greater exertion.

The other activities, like washing and ironing clothes (2.74), indicated greater difficulty and the need for support. While making the bed (1.73) and doing the shopping (1.72) seemed easier. It is however evident from these results that some participants had difficulties regarding their independence.

The results of the regression analysis presented in Table 2 explained a substantial proportion of the variance in happiness (R^2^ = 0.730, F = 21.139, *p* < 0.000). While this suggests the strong explanatory power of the specified predictors and interaction terms, the magnitude of the explained variance should be interpreted. Partial conceptual overlap among constructs such as meaning in life, perceived social support, and happiness may further contribute to shared variance.

According to the results of the diagnostic tests, the regression model was found to have a statistically strong foundation, evidenced by an average VIF of 1.45 and does not provide any evidence of multicollinearity between predictor variables (see Table 2). Furthermore, the results of the Breusch–Pagan test (*p*-value: 0.5642) indicated no significant heteroscedasticity. In addition to multicollinearity and heteroscedasticity diagnostics, the normality of the residuals was further assessed using the Shapiro–Wilk test. The results indicated that the distribution of residuals did not significantly deviate from normality (*p* > 0.05), supporting the assumption of normally distributed errors. Normality of residuals was assessed via visual inspection of P–P plots and histograms of standardised residuals. As a result, the assumption that error variance remains constant was upheld within this regression model.

Meaning in Life (MLQ) was a significant predictor of subjective well-being (PHI). MLQ demonstrated a positive and statistically significant coefficient (Coef. = 0.614, *p* = 0.039), indicating that higher perceived meaning in life was associated with higher subjective well-being.

Social support (MSPSS) also had a highly significant and strong effect on subjective well-being (Coef. = 7.695, *p* = 0.012). This effect was greater than that for meaning in life. Performance of activities of daily living (GARS) was not shown to be a direct predictor of PHI (Coef. = –0.142, *p* = 0.767). Meaning in life and social support were significant predictors, while functional ability was not.

In the analysis of interaction, MLQ#GARS appeared as significantly different from zero (Coef. = 0.013, *p* = 0.037). MSPSS#GARS was also statistically significant (Coef. = 0.084, *p* < 0.001).

**Figure 2:**
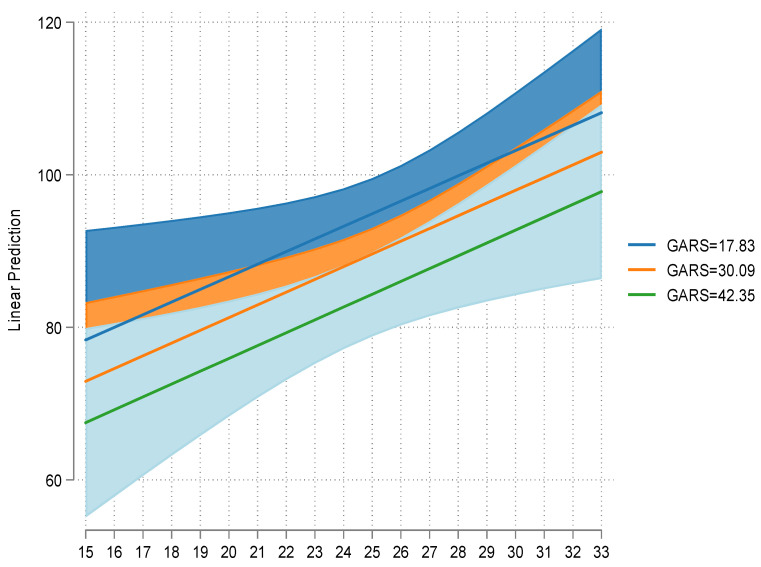
Interactive effect of MLQ and GARS on PHI.

Figure 2 depicts the major relationships among the variables, PHI, MLQ, MSPSS, and GARS, as represented by the model coefficients. The figure shows that MLQ (Meaning in Life) and MSPSS (Social Support) affect PHI directly and positively, with coefficients of 0.314 and 0.275, respectively. MLQ and MSPSS had a moderately positive relationship. GARS shows a negative relationship with PHI (−0.168).

There were strong negative associations of GARS with MLQ (–0.318) and of MLQ with MSPSS (–0.421). As shown in Figure 2, the visual model indicates that MLQ and MSPSS had a stronger influence on PHI than GARS, while GARS indirectly affects PHI by weakening participants’ psychological and social resources (Figure A1).

The graph shows a positive relationship between MLQ and PHI; as MLQ increases, the predicted value of PHI increases for all three GARS levels. This means that MLQ is associated with higher PHI scores, but the strength of this relationship varies by GARS level.

The lines are not perfectly parallel, so the graph suggests an interaction effect between MLQ and GARS. At low GARS levels, PHI starts higher and gradually increases with MLQ, while at high GARS levels, the starting point is lower, but the increase with MLQ remains clear. This indicates that GARS modulates the relationship between MLQ and PHI. The effect of MLQ on PHI is not the same for all individuals but depends on the level of GARS.

The different colored lines and shaded areas represent the predicted PHI values and their 95% confidence intervals at three GARS levels: blue for high (GARS ≥17.83), orange for mean (GARS = 30.09), and green for low (GARS = 64.36), illustrating the interaction effect with MLQ.

## 4. Discussion

This study examined the relationships between meaning in life, perceived social support, daily functioning, and happiness among older adults. The findings demonstrate that meaning in life and social support are significant predictors of well-being, while functional ability operates as a moderating factor rather than a direct determinant. The positive association between meaning in life and happiness in this study is consistent with prior research linking a stronger sense of purpose to psychological resilience and emotional well-being [5,6,7]. From a theoretical perspective, this supports eudemonic models of well-being, which emphasise meaning and purpose as central to long-term psychological health. Similarly, the strong relationship between social support and happiness aligns with extensive literature highlighting the protective role of social relationships [9,10,20,31]. These associations may reflect underlying mechanisms such as emotional reassurance, practical assistance, and reinforcement of social identity, although such pathways cannot be confirmed by the present cross-sectional design. These results are in line with the work of [13], which argues that social support acts as a buffer against the daily challenges of ageing. Although the effect of functioning as a moderator of the relationships between meaning in life, social support, and happiness was found based on observation, there is no direct behavioural or psychological evidence that those with high functional limitations use more psychosocial resources; the moderation only indicates the degree of relationship between the variables depending upon level of functioning. Thus, any interpretation should remain a report of corresponding relationships rather than inferred coping strategies.

A key contribution of the present study is the identification of functional ability as a moderating factor. The absence of a direct relationship between functional ability and happiness suggests that physical functioning alone does not determine well-being. However, the significant interaction effects indicate that the strength of the association between psychological and social resources and happiness varied according to functional limitations. This finding is consistent with the stress-buffering model, which proposes that psychosocial resources become increasingly important under conditions of vulnerability. Functional limitations may therefore be associated with greater reliance on meaning in life and social support as compensatory mechanisms. Unlike the MLQ and MSPSS, the daily activity scale (GARS) did not show a direct effect on happiness, which is consistent with several studies reporting that physical functioning is not always a direct determinant of well-being [14]. The most important finding is GARS’s moderating role. The significant interaction between GARS and MLQ, as well as between GARS and MSPSS, indicates that the association between meaning in life, social support, and happiness differed according to physical limitations. This suggests that, among individuals with higher functional limitations, psychological and social resources were more strongly associated with happiness. The finding is consistent with the literature arguing that psychological factors become more important when physical functioning declines [15,32].

Our results contribute to the current literature by providing new evidence for the role of daily activity as a moderator, a mechanism that is unexplored in studies from 2015 to 2025. While studies [33,34] emphasise the importance of daily activities for well-being, they do not test this variable as a moderator of psychological and social relationships. In this regard, our study presents a novel approach, demonstrating that GARS is not only associated with well-being but also modifies the strength of the association between MLQ and MSPSS and happiness. This has important theoretical and practical implications, as it suggests that interventions to increase meaning in life and strengthen social support may be more effective in older adults with functional limitations.

One plausible explanation is that functional limitations may restrict individuals’ access to a variety of activity-based sources of well-being, including leisure activities, employment opportunities, and physical exercise. Consequently, this restriction may be associated with greater psychological reliance on internal resources, such as the search for meaning in life, as well as on interpersonal resources, such as social support. This trend is consistent with a resource-dependency hypothesis, which posits that the significance of psychosocial resources amplifies when behavioural options become constrained.

Persons with diminished functional ability may engage in more intentional processes of meaning-making and social investment as adaptive strategies in response to their loss. This dynamic aligns with the Selection, Optimisation, and Compensation model proposed by [35].

These findings are particularly relevant for nurses, occupational therapists, geriatric care providers, and community healthcare practitioners working with older adults experiencing functional decline. Perceived social support and a sense of meaning may help identify individuals at risk of reduced quality of life despite physical health.

The present findings are also consistent with contemporary evidence on healthy ageing, resilience, and psychosocial adaptation. Recent studies suggest that older adults may maintain psychological well-being despite functional decline by relying on internal psychological resources and supportive social relationships, highlighting resilience and adaptive coping as central components of healthy ageing. Likewise, contemporary developments in Socioemotional Selectivity Theory emphasise that age-related changes in motivation promote greater investment in emotionally meaningful relationships and psychological resources, providing a theoretical basis for the moderating role of functional ability in the present study [36,37].

These findings also have important implications for healthcare practice. The present findings highlight the importance of adopting an integrated approach to healthcare delivery for community-dwelling older adults attending day-care centres. While meaning in life and social support were positively associated with well-being, these associations were not uniform, varying according to individuals’ levels of functional ability. In particular, the diminishing impact of psychosocial resources in the presence of functional limitations suggests that interventions focusing solely on social or psychological support may be insufficient for more vulnerable individuals.

From a healthcare perspective, these findings demonstrate the need for routine screening within community-based services, including day-care centres, to assess not only psychosocial factors but also functional capacity. In some settings, where such centres primarily serve recreational and social purposes, there may be limited structured approaches to supporting psychosocial well-being. This highlights an important opportunity to strengthen their role by incorporating more structured, health-oriented interventions that address both functional and psychosocial needs. Potential interventions may include social prescribing initiatives, occupational therapy programmes aimed at maintaining engagement in meaningful daily activities, structured peer-support groups, caregiver-supported self-management strategies, and community-based interventions designed to reduce social isolation.

Healthcare providers and service coordinators should therefore prioritise integrated, person-centred care models that promote engagement in daily activities while reinforcing social connections and a sense of meaning in life. This approach is consistent with international frameworks that emphasise coordinated, person-centred care as essential for improving health and well-being in ageing populations (WHO, 2025). The findings support current international healthy ageing strategies that advocate integrated, community-based, person-centred care models aimed at maintaining functional independence, psychosocial well-being, and social participation among older adults. Such approaches may enhance intervention effectiveness, optimise resource use, and support the development of more responsive and sustainable care systems for ageing populations. The results of the present study are consistent with recent findings from several studies investigating the relationship between functional ability and quality of life among older adult populations. Studies [38,39] noted that the contributions of caregivers, social support, and seniors’ self-care capabilities are critical to promoting the well-being of seniors living with multiple chronic illnesses. Their results showed that having a supportive network of family and friends, as well as having help with self-management, is vital to improving the quality of life and functionality of older adults. The present study builds upon these findings by demonstrating that, while functional ability is significant in determining an older adult’s daily ability to live independently, it also impacts the degree to which psychological meaning and social support contribute to feelings of happiness. Findings from these studies suggest that a more comprehensive approach to delivering services to seniors, addressing their physical functioning, psychosocial well-being, and supportive care relationships, is the best way to provide effective care.

The sample consisted of male participants (76.9%), which is not typical for studies involving older adults receiving community-based social services, where females are generally over-represented. This gender imbalance reflects characteristics specific to the selected day-care centres or broader regional demographic patterns; however, its underlying causes could not be determined within the scope of the present study. This limitation may affect the external validity of the findings, particularly given evidence that gender may influence the strength and direction of associations between meaning in life, social support, and subjective well-being in later life [13].

Due to the contemporaneous nature of the research design, causal inference cannot be drawn from the current observations. The reports between the constructs of life meaning, social support, the abilities available for day-to-day living, and happiness each demonstrate one-time-point or concurrent association. Therefore, reverse causation and unmeasured third-variable confounding may still apply to the present results; they can at best be viewed as associational, evidence relevant to but not providing support for the theoretical constructs outlined. Additional research in a longitudinal or experimental manner is needed to determine the directionality of the established relationships.

There are some limitations to consider when interpreting the results of this study. First, the cross-sectional nature of the survey means that any inferences made cannot be interpreted causally. Second, the choice of participants based on purposive sampling in a day centre may make it difficult to generalise the findings—many of the participants in this study may be more socially engaged than people with disabilities from the general population. Third, most participants were male. This reflects Kosovan society where males are more socially engaged outside the home. Fourth, the study did not measure some of the important covariates that could have impacted the results, including health and comorbidity. Both variables are likely to be associated with functional ability and may independently predict happiness in older adults. The exclusion of these covariates raises the possibility that the observed associations between psychosocial variables and happiness, as well as the moderation effects, are partially confounded by underlying health status. Future research should incorporate validated measures of physical health and chronic illness burden (Cumulative Illness Rating Scale for Geriatrics) to enable more robust and interpretable findings.

An additional limitation concerns potential unmeasured confounding variables. Factors such as physical health status, chronic disease burden, cognitive functioning, depressive symptoms, and socioeconomic conditions may influence both psychosocial resources and subjective well-being in older adults. The absence of these variables in the present analysis may therefore result in residual confounding, potentially affecting the observed associations and moderation effects. Future research should incorporate comprehensive health and socioeconomic indicators to provide a more robust and multidimensional understanding of the relationships under investigation.

As a result of the recruitment strategy, a selection bias may have been introduced into the study. The only people eligible for inclusion in the study were older adults who participated in community day-care centres and could independently complete the questionnaire. As a result, individuals with higher levels of social engagement, better functional capacity, or greater motivation to participate may have been over-represented. Thus, when interpreting the results of this study, consider the possibility that the relationships between meaning in life, social support, functional capacity, and happiness may not be the same for older adults who are socially isolated, institutionalised or living with more severe disabilities.

The relatively high proportion of variance explained by the regression model (R^2^ = 0.730) warrants further interpretation beyond its statistical magnitude. This value is notably higher than is typical in psychosocial research on well-being in older adults, and part of it likely reflects conceptual and measurement overlap among the study’s core constructs rather than solely the explanatory strength of functional ability as a moderator. Meaning in life, perceived social support, and happiness are theoretically related facets of subjective well-being rather than fully independent constructs: the Pemberton Happiness Index incorporates items reflecting purpose, relationships, and personal fulfilment that conceptually resemble content captured by the MLQ and MSPSS. This item-level proximity may inflate shared variance beyond what would be expected from genuinely distinct predictors. In addition, because all constructs were measured concurrently via self-report within the same cross-sectional survey, common method variance arising from shared response tendencies, social desirability, or mood at the time of assessment may further contribute to the observed R^2^. These considerations suggest that the high explained variance should be interpreted as reflecting both a meaningful substantive relationship between psychosocial resources and happiness and, at least in part, the conceptual closeness of the instruments used to measure them. Future research employing confirmatory factor analysis or multi-method assessment (combining self-report with informant ratings or behavioural indicators) would help disentangle genuine predictive relationships from construct overlap.

## 5. Conclusions

Despite extensive research on meaning in life, social support, and functional ability as predictors of well-being, few studies have examined how these factors interact. This study addresses that gap by exploring how daily functioning moderates the relationship associations between psychosocial resources and happiness in older adults, offering a more integrated understanding of well-being in later life.

The findings showed that meaning in life and social support were positively associated with happiness among older adults. While daily functioning did not directly predict happiness, it significantly moderated the relationship between meaning in life, social support, and happiness. As functional limitations increased, the associations between meaning in life, social support, and well-being became stronger, indicating a closer relationship between psychosocial resources and happiness among more functionally limited individuals. These results highlight the growing importance of psychological and social resources when physical functioning declines.

The regression model explains a substantial proportion of variance in happiness. However, the practical relevance of the moderating effects should be evaluated based on the incremental contribution of the interaction terms. Specifically, both interaction effects were statistically significant (MLQ × GARS: β = 0.013, *p* = 0.037; MSPSS × GARS: β = 0.084, *p* < 0.001), indicating that functional ability significantly moderates the associations between psychosocial resources and happiness. However, given the small magnitude of the interaction coefficients, the practical significance of these moderating effects appears limited in incremental terms beyond the main effects. These findings therefore suggest that functional ability is a theoretically meaningful moderator in the relationships between meaning in life, social support, and happiness.

This study makes an important contribution by demonstrating the moderating role of daily functioning in the associations between meaning in life, social support, and happiness. The findings carry practical implications for several groups involved in promoting healthy ageing. For clinicians and geriatric care providers, the results support incorporating brief measures of meaning in life and social support alongside standard functional assessments, since psychosocial resources appear especially important for the happiness of older adults with greater functional limitations. For community health practitioners working in day-care and social-service settings, the findings point to the value of structured programmes—such as peer-support groups, social prescribing, and activities that foster a sense of purpose—as a complement to services focused primarily on physical or recreational needs. For policymakers, the results reinforce the case for integrated, person-centred models of care for ageing populations that address functional, psychological, and social needs jointly, rather than treating physical functioning as a proxy for overall well-being.

Future research should build on these findings using longitudinal or experimental designs to clarify the direction of the observed associations and should extend the sample beyond day-care attendees to include older adults who are socially isolated, institutionalised, or living with more severe functional limitations. Incorporating objective or informant-based measures of functioning and health status alongside self-report instruments would further strengthen confidence in these findings and help determine whether targeted psychosocial interventions can meaningfully improve happiness among functionally limited older adults.

## Figures and Tables

**Table 1:** Demographic data results.

Question	Frequency	Percent
**Gender**	***N*** ** = 221**	**% = 100**
Male	170	76.9
Female	51	23.1
**Ethnicity**	***N*** ** = 221**	**% = 100**
Albanian	210	95.0
Serbian	1	0.5
Turkish	4	1.8
Bosniak	5	2.3
Rom	1	0.5
**Age Group**	***N*** ** = 221**	**% = 100**
65–70	90	40.7
71–75	73	33.0
76–81	42	19.0
82+	16	7.2
**Residence**	***N*** ** = 221**	**% = 100**
Rural	45	20.4
Urban	176	79.6
**Education**	***N*** ** = 221**	**% = 100**
No formal education	21	9.5
Primary school (ages 7–15)	64	29.0
High school	90	40.7
Bachelor’s degree	34	15.4
Master’s degree	10	4.5
Ph.D. or higher	1	0.5
Prefer not to say	1	0.5
**Marital status**	***N*** ** = 221**	**% = 100**
Married	162	73.3
Single	2	0.9
Widowed	54	24.4
Divorced	3	1.4
**Religion**	***N*** ** = 221**	**% = 100**
Catholicism/Christianity	4	1.8
Islam	208	94.1
Prefer not to say	9	4.1
**Monthly income**	***N*** ** = 221**	**% = 100**
No income	14	6.3
Less than €110	40	18.1
€111–€250	129	58.4
€251–€400	31	14.0
€401–€500	5	2.3
Over €501	2	0.9
**Source of income (Multiple response)**	***N*** **= 221**	
Pension	203	
Private investment	5	
Help from children	87	
**Lives with other family members/number living in a house?**	**Mean**	
	5.59	

**Table 2:** Results of regression analysis. ** *p* ≤ 0.01; *** *p* ≤ 0.001.

PHI	Coef.	St.Err.	t-Value	*p*-Value	95% Conf.	Interval	Sig
MLQ	0.614	0.272	2.25	0.039	0.03	1.20	**
MSPSS	7.695	3.052	2.52	0.012	1.68	13.71	**
GARS	−0.142	0.48	−0.30	0.767	−1.089	0.80	
MLQ#GARS	0.013	0.006	2.16	0.037	0.001	0.025	**
MSPSS#GARS	0.084	0.026	3.23	0.000	0.271	0.10	***
Constant	41.858	18.003	2.33	0.021	6.373	77.34	**
Mean dependent var		92.6	SD dependent var		18.103		
R-squared		0.730	Number of observations		221		
F-test		21.1	Prob > F		0.000		
Akaike crit. (AIC)		1829.8	Bayesian crit. (BIC)		1850.260		
Mean VIF = 1.45		Breusch–Pagan Test *p*-value = 0.5642			Shapiro–Wilk test, all variables *p* > 0.05		

## Data Availability

The raw data supporting the conclusions of this article will be made available by the authors on request.

## Abbreviations

The following abbreviations are used in this manuscript:

ADL	Activities of Daily Living
GARS	Groningen Activity Restriction Scale
MLQ	Meaning in Life Questionnaire
MSPSS	Multidimensional Scale of Perceived Social Support
PHI	Pemberton Happiness Index
WHO	World Health Organisation

## Appendix A

**Table A1:** Descriptive statistics of the Pemberton Happiness Index.

Domains	Item	Mean	Std Dev
General well-being	I am very satisfied with my life	7.45	2.46
	I have energy to complete my daily tasks	7.11	2.52
Eudaimonic well-being	I think my life is useful and worthwhile	7.59	2.44
	I am satisfied with myself	7.96	2.42
	My life is filled with life experiences and challenges that make me grow	6.83	2.53
	I feel very connected to the people around me	7.65	2.52
	I feel that I am able to solve most of my daily tasks	7.28	2.45
	I think that I can be myself about important things	7.6	2.35
Hedonic well-being	I enjoy small things every day	7.48	2.39
	I have many bad moments in my daily life	5.11	3.03
Social well-being	I think I live in a society that allows me to fully realise my potential.	5.86	2.81
**Satisfaction with the day before items on experienced well-being (five negative and five positive)**			
**Domains**	**Item**	**Mean**	**Std Dev**
Positive experiences	Yesterday I did something that made me proud	1.38	0.48
	Sometimes, I felt very tired/burdened	1.41	0.49
	I did something fun with someone(s)	1.31	0.46
	I was bored most of the time	1.62	0.48
	I did something that really pleased me	1.32	0.46
Negative experiences	I was worried about personal things	1.53	0.50
	I learned something interesting	1.50	0.50
	Things happened that really angered me	1.56	0.49
	I rewarded myself	1.34	0.47
	I felt disrespected by someone	1.71	0.45

**Table A2:** Descriptive statistics of the Meaning in Life.

Domains	Item	Mean	Std Dev
Presence of Meaning	I know the meaning of my life	5.68	1.27
	My life has a clear purpose	5.56	1.27
	I understand what makes my life meaningful	5.67	1.22
	I have discovered a satisfying purpose in life	5.17	1.45
	My life does not have a clear purpose	3.33	1.99
Search for Meaning	I am looking for something that makes my life meaningful	4.48	1.87
	I am always looking to find the purpose of my life	4.25	1.87
	I am always looking for something that makes my life meaningful	4.54	1.80
	I am looking for a purpose or mission for my life	4.06	1.93
	I am looking for the meaning of my life	4.07	1.91

**Table A3:** Descriptive statistics of Multidimensional Scale of Perceived Social Support.

Domains	Item	Mean	Std Dev
Family	My family really tries to help me	5.96	1.31
	I get the emotional support and help I need from my family	5.84	1.40
	I can talk about my problems with my family	5.84	1.46
	My family is willing to help me make decisions	5.74	1.41
Friends	My friends really try to help me	5.00	1.56
	I can rely on my friends when things go wrong	5.10	1.63
	I have friends with whom I can share my joys and sorrows	5.47	1.53
	I can talk about my problems with my friends	5.14	1.69
Other Significant	There is a special person who is there when I need them	5.96	1.34
	I have a special person with whom I can share my joys and sorrows	5.98	1.34
	I have a special person who is a real source of comfort for me	5.97	1.29
	There is a special person who cares about my feelings.	5.61	1.46

**Table A4:** Descriptive statistics of Groningen Activity Restriction.

Domains	Item	Mean	Std Dev
Activities of Daily Living	Can you dress yourself completely independently?	1.22	0.573
	Can you lie down and get out of bed completely independently?	1.29	0.609
	Can you get up from a chair completely independently when sitting?	1.29	0.652
	Can you wash your face and hands completely independently?	1.26	0.621
	Can you wash and dry your entire body completely independently?	1.33	0.685
	Can you sit down and get up from the toilet completely independently?	1.33	0.697
	Can you feed yourself completely independently?	1.21	0.599
	Can you move around the house completely independently (even with a cane/stick)?	1.34	0.726
	Can you go up and down stairs completely independently?	1.67	0.931
	Can you, completely independently, walk outside (even with a cane/stick)?	1.47	0.850
	Can you, completely independently, take care of your feet and toenails?	1.50	0.947
Instrumental activities of daily living	Can you completely independently prepare breakfast or lunch?	1.98	1.291
	Can you completely independently prepare dinner?	2.13	1.350
	Can you completely independently “do” household activities (for example, dusting and tidying up)?	2.25	1.416
	Can you completely independently do “heavy” household activities (for example, sweeping, cleaning windows and vacuuming)?	2.55	1.466
	Can you completely independently wash and iron clothes?	2.74	1.503
	Can you completely independently make the bed?	1.73	1.175
	Can you completely independently go shopping/do the shopping?	1.72	1.152

## References

[1] United Nations Department of Economic and Social Affairs, Population Division (2022). World Population Prospects 2022: Key Messages. https://www.un.org/development/desa/pd/sites/www.un.org.development.desa.pd/files/undesa_pd_2022_wpp_key-messages.pdf.

[2] Beard J.R., Officer A., de Carvalho I.A., Sadana R., Pot A.M., Michel J.P., Lloyd-Sherlock P., Epping-Jordan J.E., Peeters G.M.E.E.G., Mahanani W.R. (2016). The World report on ageing and health: A policy framework for healthy ageing. Lancet.

[3] Steptoe A., Deaton A., Stone A.A. (2015). Subjective well-being, health, and ageing. Lancet.

[4] Joshanloo M., Blasco-Belled A. (2023). Reciprocal associations between depressive symptoms, life satisfaction, and eudaimonic well-being in older adults over a 16-year period. Int. J. Environ. Res. Public Health.

[5] Alimujiang A., Wiensch A., Boss J., Fleischer N.L., Mondul A.M., McLean K., Mukherjee B., Pearce C.L. (2019). Association between life purpose and mortality among US adults older than 50 years. JAMA Netw. Open.

[6] Hedberg P., Brulin C., Aléx L., Gustafson Y. (2011). Purpose in life over a five-year period: A longitudinal study in a very old population. Int. Psychogeriatr..

[7] García-Alandete J. (2015). Does meaning in life predict psychological well-being?. Eur. J. Couns. Psychol..

[8] Hervás G., Vázquez C. (2013). Construction and validation of a measure of integrative well-being in seven languages: The Pemberton Happiness Index. Health Qual. Life Outcomes.

[9] Santini Z.I., Jose P.E., Cornwell E.Y., Koyanagi A., Nielsen L., Hinrichsen C., Meilstrup C., Madsen K.R., Koushede V. (2020). Social disconnectedness, perceived isolation, and symptoms of depression and anxiety among older Americans (NSHAP): A longitudinal mediation analysis. Lancet Public Health.

[10] Courtin E., Knapp M. (2017). Social isolation, loneliness and health in old age: A scoping review. Health Soc. Care Community.

[11] Holt-Lunstad J., Smith T.B., Layton J.B. (2010). Social relationships and mortality risk: A meta-analytic review. PLoS Med..

[12] Santini Z.I., Koyanagi A., Tyrovolas S., Mason C., Haro J.M. (2015). The association between social relationships and depression: A systematic review. J. Affect. Disord..

[13] Cheng S.T., Fung H.H., Chan A.C. (2008). Living status and psychological well-being: Social comparison as a moderator in later life. Aging Ment. Health.

[14] Feng Z., Cramm J.M., Nieboer A.P. (2020). Social participation is an important health behaviour for health and quality of life among chronically ill older Chinese people. BMC Geriatr..

[15] Lee S., Urban-Wojcik E.J., Charles S.T., Almeida D.M. (2022). Rich and balanced experiences of daily emotions are associated with activity diversity across adulthood. J. Gerontol. B Psychol. Sci. Soc. Sci..

[16] Cohen S., Wills T.A. (1985). Stress, social support, and the buffering hypothesis. Psychol. Bull..

[17] Havighurst R.J. (1961). Successful aging. Gerontologist.

[18] Adams K.B., Leibbrandt S., Moon H. (2011). A critical review of the literature on social and leisure activity and wellbeing in later life. Ageing Soc..

[19] Bigonnesse C., Chaudhury H. (2020). The landscape of “aging in place” in gerontology literature: Emergence, theoretical perspectives, and influencing factors. J. Aging Environ..

[20] Huxhold O., Fiori K.L., Windsor T.D. (2020). The dynamic interplay of social network characteristics, subjective well-being, and health: The costs and benefits of socio-emotional selectivity. Psychol. Aging.

[21] Baron R.M., Kenny D.A. (1986). The moderator–mediator variable distinction in social psychological research: Conceptual, strategic, and statistical considerations. J. Personal. Soc. Psychol..

[22] Newman D.B., Nezlek J.B., Thrash T.M. (2020). The dynamics of searching for meaning and presence of meaning in daily life. J. Personal..

[23] Cohen J., Cohen P., West S.G., Aiken L.S. (2001). Applied Multiple Regression/Correlation Analysis for the Behavioral Sciences.

[24] Faul F., Erdfelder E., Buchner A., Lang A.G. (2009). Statistical power analyses using G*Power 3.1: Tests for correlation and regression analyses. Behav. Res. Methods.

[25] Steger M.F., Frazier P., Oishi S., Kaler M. (2006). The Meaning in Life Questionnaire: Assessing the Presence of and Search for Meaning in Life. J. Couns. Psychol..

[26] Dahlem N.W., Zimet G.D., Walker R.R. (1991). The multidimensional scale of perceived social support: A confirmation study. J. Clin. Psychol..

[27] Zimet G.D., Dahlem N.W., Zimet S.G., Farley G.K. (1988). The multidimensional scale of perceived social support. J. Personal. Assess..

[28] Kempen G.I.J.M., Miedema I., Ormel J., Molenaar W. (1996). The assessment of disability with the Groningen Activity Restriction Scale. Conceptual framework and psychometric properties. Soc. Sci. Med..

[29] Suurmeijer T.P., Doeglas D.M., Moum T., Briançon S., Krol B., Sanderman R., Guillemin F., Bjelle A., Heuvel W.J.V.D. (1994). The Groningen Activity Restriction Scale for measuring disability: Its utility in international comparisons. Am. J. Public Health.

[30] (1997). Convention for the Protection of Human Rights and Dignity of the Human Being with Regard to the Application of Biology and Medicine (Oviedo Convention).

[31] Burholt V., Winter B., Aartsen M., Constantinou C., Dahlberg L., Feliciano V., Gierveld J.D.J., Van Regenmortel S., Waldegrave C. (2020). A critical review and development of a conceptual model of exclusion from social relations for older people. Eur. J. Ageing.

[32] Tarrant A., Way L., Ladlow L. (2023). Men and Welfare.

[33] Bu F., Steptoe A., Mak H.W., Fancourt D. (2021). Time use and mental health in UK adults during an 11-week COVID-19 lockdown: A panel analysis. Br. J. Psychiatry.

[34] Azcurra D.J.L.S. (2012). A reminiscence program intervention to improve the quality of life of long-term care residents with Alzheimer’s disease: A randomized controlled trial. Rev. Bras. Psiquiatr..

[35] Baltes P.B., Baltes M.M. (1990). Psychological perspectives on successful aging: The model of selective optimization with compensation. Successful Aging: Perspectives from the Behavioral Sciences.

[36] Carstensen L.L., Isaacowitz D.M., Charles S.T. (1999). Taking time seriously: A theory of socioemotional selectivity. Am. Psychol..

[37] Simons M., Reijnders J., Janssens M., Lataster J., Jacobs N. (2023). Positive affect as mediator: The socioemotional selectivity theory applied to the association between bonding social capital and wellbeing in later life. J. Soc. Personal. Relatsh..

[38] Adëraj S., Saurini M., Mazzotta R., Gara E., Taçi D., Arapi A., Bernalte-Martí V., Stievano A., Vellone E., Rocco G. (2025). Caregiver contribution to patient self-care and associated variables in older adults with multiple chronic conditions living in a middle-income country: Key findings from the ‘SODALITY-AL’ observational study. Nurs. Rep..

[39] Gara E., Shuleta-Qehaja S., Gara E. (2024). Nurses’ attitudes to care of the elderly. Multidiscip. Sci. J..

